# Comparative analysis of milk bacteria by two MALDI-MS systems

**DOI:** 10.1038/s41598-025-21019-0

**Published:** 2025-10-24

**Authors:** Jagoda Pałczyńska, Oleksandra Pryshchepa, Agnieszka Ludwiczak, Adrian Arendowski, Ewelina Sibińska, Aleksandra Radtke, Paweł Pomastowski

**Affiliations:** 1 Centre for Modern Interdisciplinary Technologies, Nicolaus Copernicus University, Toruń, Poland; 2 Department of Inorganic and Coordination Chemistry, Faculty of Chemistry, Nicolaus Copernicus University, Toruń, Poland; 3 Department of Immunology, Faculty of Biological and Veterinary Sciences, Nicolaus Copernicus University, Toruń, Poland; 4 Department of Inorganic and Analytical Chemistry, Faculty of Chemistry, Rzeszów University of Technology, Rzeszów, Poland

**Keywords:** Mass spectrometry, Milk bacteria, MALDI-TOF MS, Microorganisms, Mass spectrometry, Metabolomics, Microbiology techniques, Chemistry

## Abstract

This study presents a comparative analysis of bacterial strains isolated from raw milk using two. MALDI-TOF MS systems: the Bruker Microflex LT Biotyper and the newer Zybio EXS2600 Ex-Accuspec. A total of 1,130 isolates were identified using the in-tube extraction method. The aim of the study was to assess identification accuracy at the genus and species levels. Both systems demonstrated high performance: the Bruker system identified 94.6% of the isolates to at least the genus level, and the Zybio system identified 91.3%. Both MALDI systems showed a high level of agreement at the species level - approximately 75% of Bruker identifications matched those obtained with the Zybio system. In the remaining ~ 25% of cases, discrepancies were observed at both the species and genus levels. The results indicate that both systems offer a comparable level of effectiveness for routine use. in microbiological diagnostics.

**Supplementary Information:**

The online version contains supplementary material available at 10.1038/s41598-025-21019-0.

## Introduction

The MALDI-TOF mass spectrometry technique has in recent years introduced a breakthrough in methods for the identification of microorganisms. Initially, it revolutionized clinical diagnostics with its fast, simple, reliable and precise identification of microorganisms. Nowadays, this technique, is also gaining increasing interest in the identification of environmental bacteria including food microbiology^1,2^ Control of the raw milk microbiome is crucial to ensure high quality and safety of dairy products. The composition of the raw milk microflora not only influences the organoleptic and sensory properties of the food produced, but can also negatively affect its quality and shelf life. In addition, the presence of pathogens such as *L. monocytogenes*, *E. coli*, or *S. aureus*, among others, can pose significant health risks^3^.

The MALDI-TOF MS technique uses soft ionization to capture a microorganism’s characteristic protein profile, which is then compared with reference spectra found in commercially available databases. On the basis of the obtained spectra, it is possible to identify the microorganism at species and/or genus level. Compared to other identification methods such as biochemical assays or 16 S rDNA sequencing, mass spectrometry is less time-consuming, less labour-intensive, and its basic operation is relatively straightforward^4,5^.

Currently, mass spectrometry is widely used for the identification of microorganisms, with commercially available systems such as the MALDI Biotyper (Bruker Daltonics, Germany) and VITEK MS (bioMérieux, France) representing the most established platforms^2,6^. These systems have been the subject of numerous comparative studies evaluating their effectiveness in microbial identification^7–9^ also in the analysis of food microbiota^10^. Recently, there has been an increase in the variety of MALDI-TOF MS systems available as a result of the launch of new products by the Chinese company Zybio^11^. Their application in the identification of microorganisms compared to leading systems has not yet been widely described in the literature. Regarding the EXS2600 system (Zybio), four articles have been published so far on the analysis of clinical isolates^12,13^, one on dairy products^14^ and diesel fuels^15^. The few studies highlight a large gap in fully assessing the functionality and usefulness of the EXS2600 system, especially outside the clinical setting.

This study evaluated the effectiveness of microbial identification using two commercial MALDI-TOF MS systems for the analysis of microorganisms isolated from raw milk samples. The widely used Bruker Microflex LT system was compared with the less well-known Zybio EXS2600 system, which is not commonly applied on the European market. The aim of the analysis was to compare the performance of both devices in bacterial identification using a sample preparation protocol involving bacterial protein extraction.

## Materials and methods

In the study, a total of 1130 microbial strains isolated from raw milk samples collected directly from animals between 2022 and 2023 were analyzed. The milk samples are originated from farm located in the Kuyavian-Pomeranian Voivodeship (Poland) and were collected from each udder quarter separately into sterile Falcon tubes by hand-stripping directly before the morning milking. Before milking, the udders were pre-cleaned with disposable udder wipes. For microbial isolation, raw milk samples were serially diluted in peptone water (to 10⁻²), and 100 µl of each dilution was spread onto agar plates. The cultures were incubated at 37 °C for 24–48 h under both aerobic conditions and CO₂-enriched atmosphere (5%). After incubation, morphologically distinct colonies were selected and subcultured onto fresh media to obtain pure cultures. The isolates were stored at − 80 °C using the Microbank system (Pro-Lab Diagnostics) for further analysis. Before the analysis strains were cultured on Tryptic Soya Agar (TSA) and incubated under aerobic conditions at 37 °C for 24 h.

### Analysis of samples by MALDI-TOF MS

Protein extraction was performed according to the standard formic acid/acetonitrile protocol recommended by the manufacturer (Bruker Daltonics). The prepared extracts were applied in a volume of 1 µl to a steel 96-spot plate and left to dry. The sample was then coated with 1 µL of a matrix solution – alpha-cyano-4-hydroxycinnamic acid (HCCA, 10 mg/mL) dissolved in standard solvent (50% acetonitrile, 47.5% HPLC-grade water, and 2.5% trifluoroacetic acid) – and again left to dry at room temperature. Subsequently, the plate was placed in MALDI mass spectrometers. Due to the compatibility of the two companies’ plates, a single MALDI plate was used. Therefore, bacteria from the same colony were applied onto two separate spots on the same plate—one for each system—ensuring a direct comparison of equivalent samples.

#### Sample analysis using the Bruker daltonics system

The prepared targets were analysed in automated mode with a Microflex LT MALDI-TOF MS mass spectrometer (Bruker Daltonics GmbH, Bremen, Germany) in positive linear mode using a 60 Hz nitrogen laser (λ = 337 nm), in the mass range of 2000–20000 *m/z*. Spectra were recorded using FlexControl version 3.4 and identified using MBT Compass version 4.1.100 (database: 10830 entries). The Bruker Bacterial Test Standard (BTS, Bruker Daltonics) was used as calibrator. The spectra were analysed following the description in the article by Maślak et al^12^.

#### Sample analysis using the Zybio system

The prepared targets were also analysed by automatic mode with an EXS2600 MALDI-TOF MS mass spectrometer (Zybio Inc., Chongqing, China) in positive linear mode using a 60 Hz nitrogen laser (λ = 337 nm), in the mass range of 2000–20000 *m/z*. Spectra were recorded and identified using System Ex-Accuspec version V1 software (database: ca. 15,000 entries). The Microbiology Calibrator (Zybio Inc., Chongqing, China) was used as the calibrator.

### Statistical analysis of the obtained identification results

Data analysis and chart generation were conducted using the PS IMAGO PRO 9.0 package (Predictive Solutions, Poland) and Python (version 3.8) with the Pandas library (version 1.2.0) and Matplotlib (version 3.4.0). A Z-test was applied to demonstrate differences between proportions at various identification levels between the Bruker and Zybio systems. The non-parametric Kruskal-Wallis test was used to demonstrate statistically significant differences between mean scores within different bacterial classes for the two MALDI systems. The identification levels were determined based on the manufacturer’s recommendations according to the following score values: a score of 2.000 or above signified species-level identification, a score between 1.700 and 1.999- the genus level and a score below 1.700 was considered a failure in identification.

## Results

### Comparative identification by MALDI-TOF MS using two systems Zybio EXS2000 and Bruker

**Fig. 1:**
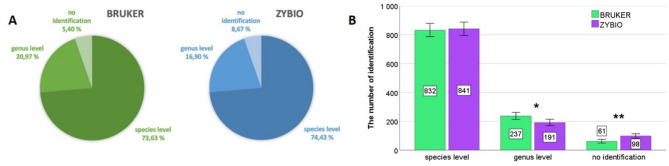
**(A)** Percentage of identification accuracy based on results for the two tested MALDI **(B)** Comparison of identification levels for two MALDI systems (Bruker and Zybio). Asterisks indicate levels of statistical significance based on the z-test results as follows: *p < 0.05, **p < 0.01. Error bars represent 95% confidence intervals.

A total of 1130 microorganisms isolated from raw milk samples were analyzed using two MALDI-TOF MS systems: Bruker Biotyper and Zybio EXS2000. Both systems demonstrated comparable species-level identification performance, with Bruker identifying 73.63% of isolates and Zybio 74.43%. However, differences were observed at the genus-only level (i.e., cases identified to genus but not to species) and in the number of unidentified cases, as shown in Fig. 1A and B. Bruker achieved a higher percentage of genus-only identifications and a lower percentage of unidentified isolates. These differences were statistically significant for genus-only identifications (*p* = 0.0135) and unidentified cases (*p* = 0.0023), but not for species-level identifications (*p* = 0.666) (Fig. 1B). In both systems, Gram-positive bacteria predominated (Supp. Figure 1).

**Fig. 2:**
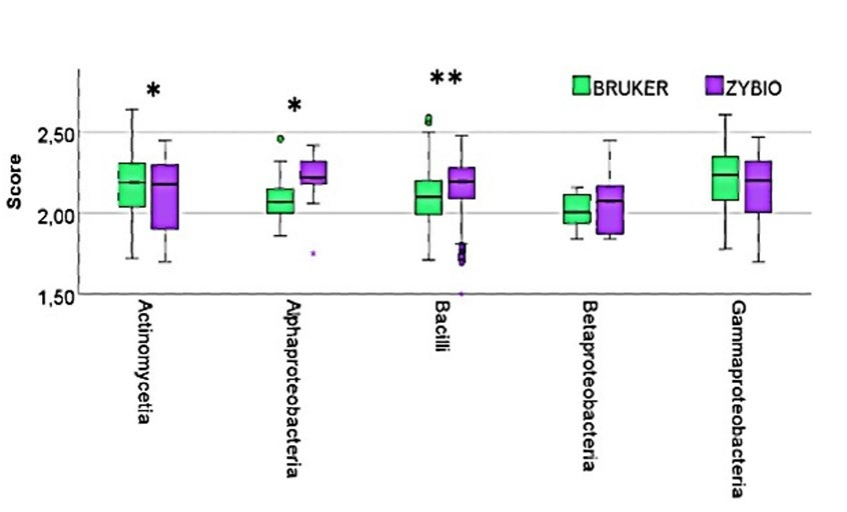
Distribution of score values for identified bacterial classes by two MALDI systems. Asterisks indicate levels of statistical significance between median based on the Kruskal-Wallis test results as follows: *p < 0.05, **p < 0.01.

The systems also differed in terms of identification reliability, expressed as the Score value. The mean scores were similar (Bruker: 2.064; Zybio: 2.098), but greater variability in Score values was observed in the Zybio system, particularly within the *Actinomycetia*, *Betaproteobacteria*, and *Gammaproteobacteria* classes. The Kruskal–Wallis test revealed statistically significant differences in Score values for the *Actinomycetia* (*p* = 0.0306), *Alphaproteobacteria* (*p* = 0.0225), and *Bacilli* (*p* < 0.001) classes (Fig. 2).

**Fig. 3:**
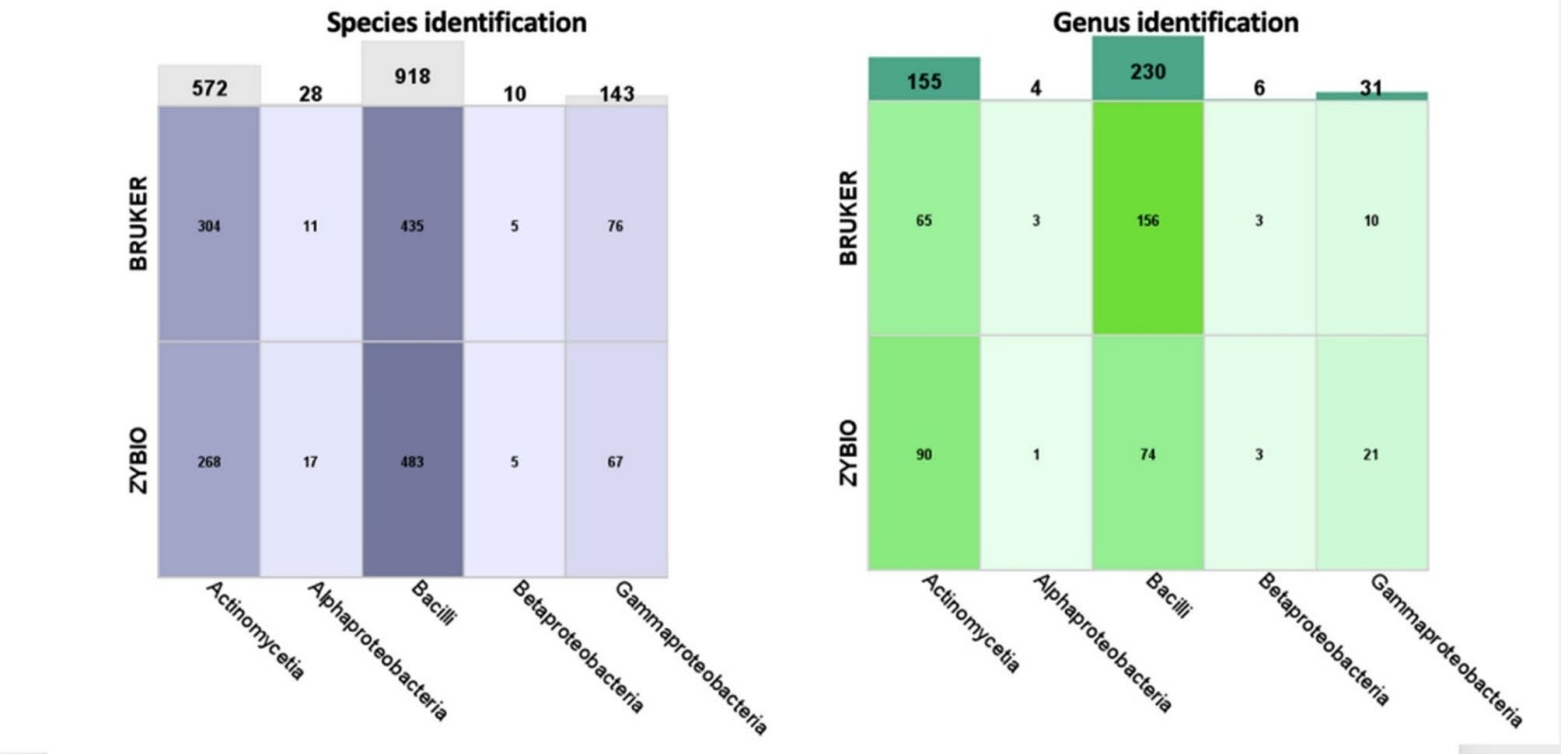
Comparison of the number of species-level (a) and genus-level (b) identifications for two MALDI systems, Bruker and Zybio. Color intensity corresponds with the number of samples identified at the respective level by each MALDI system.

Both the Zybio and Bruker systems showed differences in the identification of microorganisms at the genus-only and species levels (Fig. 3). The highest proportion of unambiguous identifications was recorded for the *Betaproteobacteria* class. Bruker more frequently identified bacteria from the *Actinomycetia* and *Gammaproteobacteria* classes, with higher Score values, while Zybio more effectively identified microorganisms from the *Alphaproteobacteria* and *Bacilli* classes. Zybio also enabled the identification of a greater number of microorganisms limited to the genus level within selected classes.

**Fig. 4:**
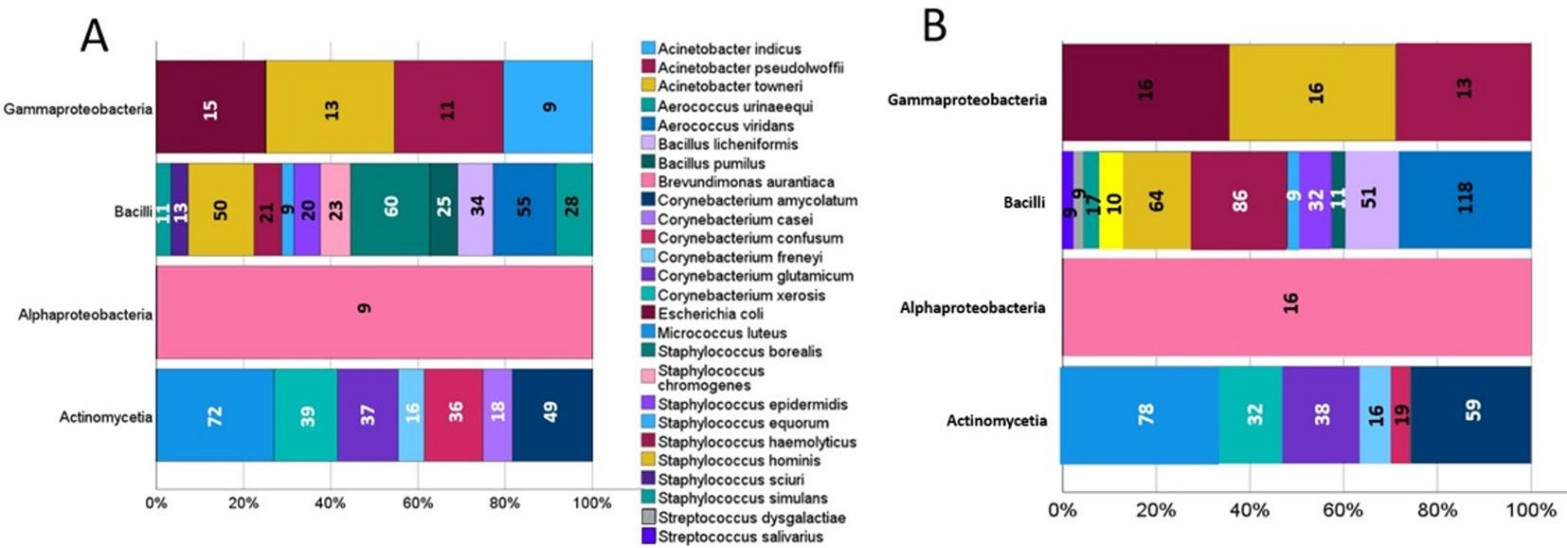
Comparison of species MALDI identification categorized by classes of identified microorganisms. The chart presents bacterial species identified by the Bruker **A.** and Zybio **B**. systems, including only those taxa that constituted more than 1% of the total number of identified microorganisms. The quantities of identified species representing each class are marked on the graph.

The most diverse class was *Bacilli*, comprising 12 and 11 species identified by Bruker and Zybio, respectively (Fig. 4). Bruker most frequently detected *Staphylococcus borealis*, *Staphylococcus hominis*, and *Aerococcus viridans*, while Zybio most often identified *Staphylococcus haemolyticus*, *Staphylococcus hominis*, and *Aerococcus viridans*. In the *Alphaproteobacteria* class, both systems consistently identified *Brevundimonas aurantiaca*, and in *Actinomycetia* – *Micrococcus luteus* and *Corynebacterium amycolatum*, although with varying frequencies. Discrepancies were also observed in the identification of the genera *Acinetobacter*, *Streptococcus*, and *Staphylococcus*.

Both MALDI systems demonstrated a high level of agreement at the species level—615 out of 832 Bruker identifications matched those obtained with Zybio. In the remaining ~ 25% of cases, discrepancies were observed at both the species level (Supp. Figure 2A) and the genus-only level (Supp. Figure 2B). The most pronounced differences involved *Aerococcus urinaeequi* (Bruker), which was classified by Zybio as *Aerococcus viridans* or *Aerococcus* spp., and *Staphylococcus borealis* (Bruker), which Zybio identified as *Staphylococcus haemolyticus*, *Staphylococcus* spp., or left unidentified. In more than half of the discordant cases, Zybio provided identification only at the genus level or failed to identify the organism at all, whereas Bruker assigned a species-level identity (Supp. Figure 2B). This was observed, for example, with *Acinetobacter indicus*, *Neisseria subflava*, *Corynebacterium casei*, and several *Staphylococcus* species.

**Fig. 5:**
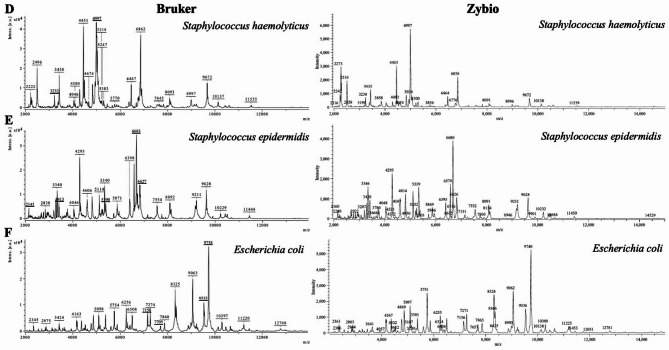
Set of exemplary MALDI-TOF MS spectra obtained for selected isolates using the Bruker system (left) and the Zybio system (right). Panels D–F show concordant species-level identifications across both systems.

**Fig. 6:**
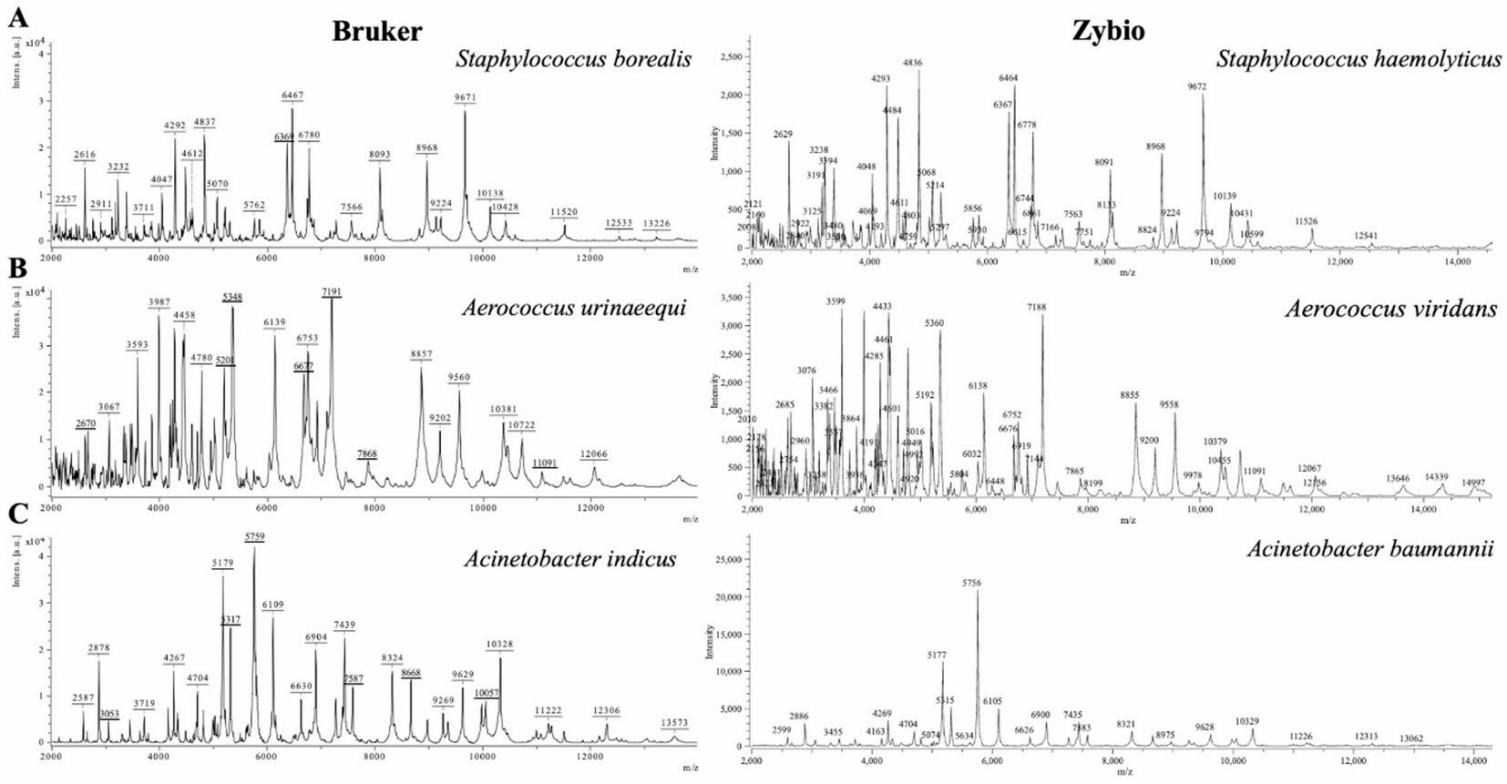
Overview of representative MALDI-TOF MS spectra obtained for selected isolates using the Bruker system (left) and the Zybio system (right). A–C: examples of discrepancies in species-level identification.

Representative MALDI-TOF MS spectra are illustrated in Figs. 5 and 6 to exemplify the comparative performance of the two systems (Bruker and Zybio). Figure 5 shows spectra of isolates consistently identified at the species level by both instruments, including *S. haemolyticus*, *S. epidermidis*, and *E. coli*. In contrast, Fig. 6 presents spectra of isolates for which divergent identifications were obtained: Bruker classified them as *S. borealis*, *A. urinaeequi*, and *A. indicus*, whereas Zybio identified the same isolates as *S. haemolyticus*, *A. viridans*, and *A. baumannii*. Notably, the majority of high-intensity peaks are conserved between the systems, while it can be observed that differences are more pronounced among low-intensity signals. The observed differences in spectral profiles and final identifications are most likely related to discrepancies in the algorithms used for spectrum acquisition and processing, as well as in the algorithms applied to assess spectral similarity for species-level classification.

## Discussion

The introduction of MALDI technology has revolutionised the identification of microorganisms in clinical laboratories, offering an alternative to traditional biochemical tests or sequencing-based methods through an approach focusing on proteomic analysis. The new method has attracted great interest due to its significantly faster and simpler identification and also its ability to process large numbers of samples simultaneously. The popularity of this technology has contributed to the rapid development of MALDI, with many companies worldwide offering mass spectrometry for the identification of microorganisms.

In a study conducted by our team, a comparison was made between two commercially available MALDI systems in the context of their effectiveness in identifying micro-organisms isolated from raw milk samples. The systems used in the study were the well-known and widely described Bruker Microflex LT, which was the first in Europe approved for clinical use. In contrast, the second system, Zybio’s EXS2600, is relatively new to the market, resulting in the limited availability of scientific literature on its applications, especially in comparative analyses. Our study provides important data in the context of comparing the performance of the two systems in the analysis of bacterial isolates obtained from environmental samples which is particularly relevant given that MALDI technology was originally designed for the analysis of clinical samples.

In this study, a protein extraction method was used to identify microorganisms. This method allows for more precise identification results, especially in the case of Gram-positive bacteria^16,17^, due to their thick and complex cell wall structure, which may limit effective protein release in standard analysis. Similarly, difficulties arise for microorganisms derived from environmental samples, which may be characterised by additional changes in cell wall structure as a result of adaptation to the surrounding environment. Importantly, the protein extraction method yields higher quality spectra, which increases the chances of correctly matching them to available databases^18^. In our case, Gram-positive bacteria accounted for more than 80% of the microorganisms identified, so the use of extraction enabled reliable results to be obtained.

The results we obtained clearly show the high efficiency of both instruments in identification using the automatic mode. The Bruker system correctly identified at least to genus level 94.6% of the isolates analysed and the second system, Zybio, 91.3%.

Similar results were reported by G.Czeszewska-Rosiak et al.^14^, who analyzed isolates originating from dairy products such as milk, buttermilk, and dairy wastewater. Both systems demonstrated high effectiveness at least at the genus level, 100% of isolates were identified using the Bruker system and 96% using the Zybio system. Instead, the species identification rate was 66,8% and 76,0% respectively. Importantly, in contrast to our analyses, as many as 78% of the microorganisms were Gram-negative bacteria. In both studies, the research material consisted of dairy products, and the results confirmed the high effectiveness of MALDI-TOF MS systems in identifying microorganisms associated with this type of sample regardless of whether the dominant group comprised Gram-positive bacteria, as in our analyses, or Gram-negative bacteria, as in the aforementioned study. This is particularly relevant in the context of veterinary diagnostics and food safety, where rapid and reliable identification of microorganisms is crucial both for animal health and the quality of dairy products. However, only 196 isolates were analyzed, and this limited number may not provide a fully representative picture, as the dataset is statistically underpowered.

Instead, in the study by E.Sibińska et al.^12^ a substantially larger number of isolates was analyzed, comprising 1,979 clinical bacterial isolates obtained from the urine of patients with prostate cancer. The comparable findings were obtained. In their study, both systems demonstrated high identification accuracy, with the Bruker system achieving 96% and the Zybio system 92% at the genus level, as well as 75% and 82% at the species level, respectively. Similar observations were reported by K.Dichtl et al.^13^, who evaluated the utility of MALDI-TOF MS systems in clinical diagnostics, pointing to the comparable effectiveness of Bruker and Zybio in identifying microorganisms isolated from patients. When relating these findings to our results, it should be noted that, although milk is also a biological sample, our study revealed a higher proportion of environmental and potentially opportunistic bacteria, which are of greater relevance in the context of dairy product quality.

Different results were reported by A.Ludwiczak et al.^15^, who analyzed bacterial isolates derived from fuel samples. In their study, the Zybio system achieved a higher proportion of species-level identifications (48% vs. 33%) and a lower percentage of unidentified isolates compared with the Bruker system. Unlike our analyses, in which both systems demonstrated comparable species-level accuracy, the study by A. Ludwiczak et al. indicated a clear advantage of Zybio. As emphasized by the authors, the origin of isolates may influence the generated mass spectra and, consequently, their matching to reference databases. Bacteria isolated from fuels may produce additional proteins and metabolites associated with adaptation to specific environmental conditions, thereby altering their proteomic profile and hindering identification. In the case of fuel-derived strains—often representing microorganisms that are rarely included in commercial libraries—this may have contributed to the relatively high proportion of unidentified isolates observed in both systems. These findings underscore the importance of continuously expanding reference databases with spectra from microorganisms originating from diverse environments, which is essential for improving the performance of MALDI-TOF MS in both clinical and environmental diagnostics.

Statistically significant differences were observed in genus-only identification and the number of samples not identified with an advantage for the Bruker system. It identified more samples at genus level and had a lower percentage of unidentified samples, which is similar to the results obtained by G.Czeszewska-Rosiak^19^who analysed swabs from oncology patients and hospital surfaces as well as E.Sibińska et al^12^. who identified microorganisms isolated from the urine of patients with prostate cancer.

Our results indicate that the Zybio system shows a higher variance in score value for the classes *Actinomycetia*, *Betaproteobacteria* and *Gammaproteobacteria*. Therefore, the performance of the Zybio system in identifying species in these classes is less consistent compared to the Bruker system. The higher variance of the results may suggest that the Zybio system may be less reliable in consistently identifying species in these classes of bacteria. The Bruker system, with its lower variance, appears to provide more stable and consistent identification results. The differences in point values may be due to the different way reference spectra are generated in the corresponding databases. As reported by E. Sibinska et al.^12^ in the case of the Zybio system, for a signal to be included in the database it must be repeated in at least 50% of the collected spectra. In the case of the Bruker system, this threshold is less rigorous at 25%. This is therefore not without impact on the point values obtained. This underlines the enormous influence of the database design and the signal criteria of the individual systems on the results obtained.

Systems have shown differences in performance and efficiency in identifying bacterial classes at both species and genus level. The choice of MALDI system may depend on the specific bacterial classes of interest and the level of identification required (species or genus). Our results indicate that each system has a class-dependent efficiency, which can help users select the right system for their specific needs. The Bruker system for more accurate for the identification of bacteria from the classes *Actinomycetia* and *Gammaproteobacteria.* Conversely, the Zybio system may be preferred for identifying bacteria from the classes *Alphaproteobacteria* and *Bacilli*, as well as for broader genus-level identifications within *Actinomycetia* and *Gammaproteobacteria*. The results suggest that both systems can be used complementarily, depending on research needs.

Another important aspect revealed by our study is the discrepancy in the number of species identified by the two systems. For example, in the case of bacteria of the genera *Staphylococcus sp.* or *Acinetobacter sp.*, there are noticeable differences in the number of most frequently identified species in each bacterial class. Furthermore, approximately 25% of the identifications (of microorganisms identified to the species level) showed discrepancies at the species or genus level between the two MALDI systems. A similar phenomenon was reported by G.Czeszewska-Rosiak et al.^14^, with discrepancies reached approximately 26%, and by E.Sibińska et al.^12^, where the rate was around 10.5%. These findings indicate that differences between the systems are not incidental but represent a recurring feature of comparative analyses, which further underscores the need for continuous improvement of reference databases and matching algorithms. For example, Fig. 5 presents spectra of selected isolates that were identified differently by the Bruker and Zybio systems, whereas Fig. 6 shows spectra of isolates consistently classified by both systems. Despite the overall similarity of the main signals, differences in database coverage and matching criteria may lead to discrepancies in identification.

An important factor influencing these discrepancies is the origin and number of strains included in the reference spectrum databases of both systems. In the case of the Zybio device, the reference database is based on clinical isolates from China, whereas the Bruker system is based mainly on European strains, including standard ATCC and DSMZ strains. Some of the discrepancies in our results can be attributed to the absence of specific strains in the Zybio system database. An example of discrepancies in identification is the pair *Staphylococcus borealis* and *Staphylococcus haemolyticus*, where *S. borealis* does not appear in the Zybio system database. In contrast, pairs such as, for example, *Staphylococcus petrasii* and *Staphylococcus epidermidis*, *Staphylococcus arlettae* and *Staphylococcus equorum*, *Streptococcus canis* and *Streptococcus dysgalactiae*, *Acinetobacter indicus* and *Acinetobacter baumannii*, and *Aerococcus viridans* and *Aerococcus urinaequi*, suffered from species-level matching errors despite the presence of these species in the databases. Although some studies clearly indicate that MALDI can be used to discriminate between closely related bacterial strains sometimes with greater success than 16 s rDNA as shown by Anderson et al.^20^ using the example of bacteria of the genus *Lactobacillus*, our results showed that errors can occur. The accuracy of identification is highly dependent on the quality and quantity of spectra in reference databases, which emphasises the need to regularly update them with new data including environmental strains. Additionally, the use of identification based on the bacterial proteome may not always be sufficient to distinguish between closely related strains. This is due to the very similar composition of ribosomal proteins, especially in highly related bacteria. Our observations indicate that the incorporation of new complementary analytical methods may be necessary for correct species differentiation, with bacterial lipid analysis being one potential approach. Lipids, alongside proteins, are key structural and functional components of cells, contributing to membrane architecture, signal transduction, energy storage, and cell recognition mechanisms. Due to their functional diversity, bacterial lipids may serve as valuable biomarkers in mass spectrometry-based identification^2^.

Several recent studies have demonstrated the potential of lipid ‘fingerprinting’ by MALDI-TOF-MS for differentiating bacterial species^21^, including *Bacillus*^22^ and *Mycobacterium*^23^ species as well as closely related strains such as *E. coli* and *Shigella spp*^24,25^. Moreover, alternative lipid profiling techniques using nanostructures, such as CaO or silver nanostructures, have shown promising results in differentiating bacterial species including drug-resistant *E.coli* strains^26^. Among these, nanostructure-assisted laser desorption/ionization (NALDI) is gaining increasing attention due to its ability to analyze low-molecular-weight compounds without the need for organic matrices, making it a potential complement to classical proteomic methods^2,27^. The parallel analysis of the proteome and lipidome may thus provide a more comprehensive strategy for microbial identification, particularly in cases where protein-based approaches alone are insufficient. Thus, in the next stages of our research, we plan to apply NALDI-based lipid profiling to assess its utility as a complementary method for species differentiation.

## Conclusions

Our study compared two MALDI systems - Bruker Microflex LT Biotyper and Zybio EXS2600 Ex-Accuspec - in identifying strains isolated from raw milk samples. The results obtained indicate the high efficiency of both instruments in identification. The discrepancies in the results suggest the need to further develop the existing databases of reference spectra and to search for new complementary methods for identification and differentiation of microorganisms.

## Supplementary Information

Below is the link to the electronic supplementary material.

Supplementary Material 1

## Data Availability

The comparative identification table obtained using two MALDI-TOF MS systems is part of this dataset available in the RepOD repository: Pryshchepa, Oleksandra; Pałczyńska, Jagoda; Ludwiczak, Agnieszka; Arendowski, Adrian; Sibińska, Ewelina; Pomastowski, Paweł, 2025, “LDI-MS analysis of bacteria isolated from milk”, [https://doi.org/10.18150/HHX375](https:/doi.org/10.18150/HHX375).
