# Supplementary material for: Comparative analysis of milk bacteria by two MALDI-MS systems
Source: Sci Rep. 2025 Oct 24;15:37206. doi: 10.1038/s41598-025-21019-0 (PMC12552733; doi:10.1038/s41598-025-21019-0)
Supplement: Supplementary file 1 — Supplementary Material 1 [file 41598_2025_21019_MOESM1_ESM.docx]

**Supplementary materials**


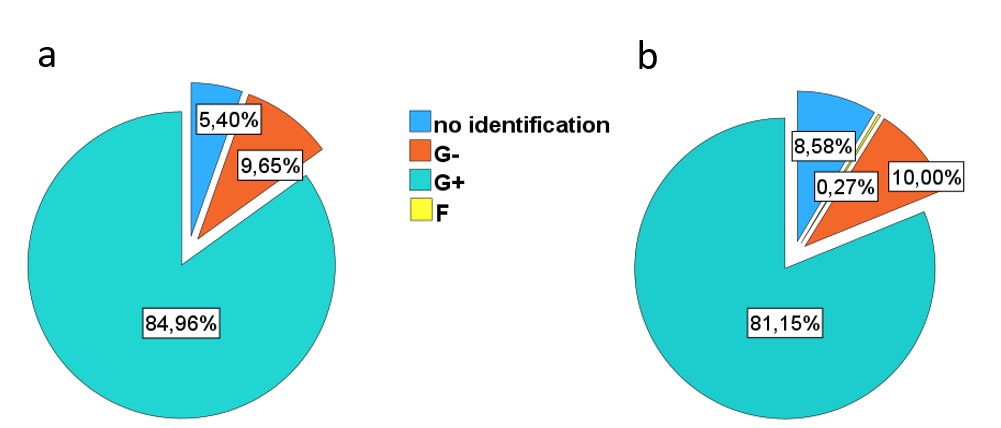


**Supplementary Figure 1.** The distribution of G+, G-, and unidentified gram type of bacteria according to **Bruker** (a) and **Zybio** (b) identification


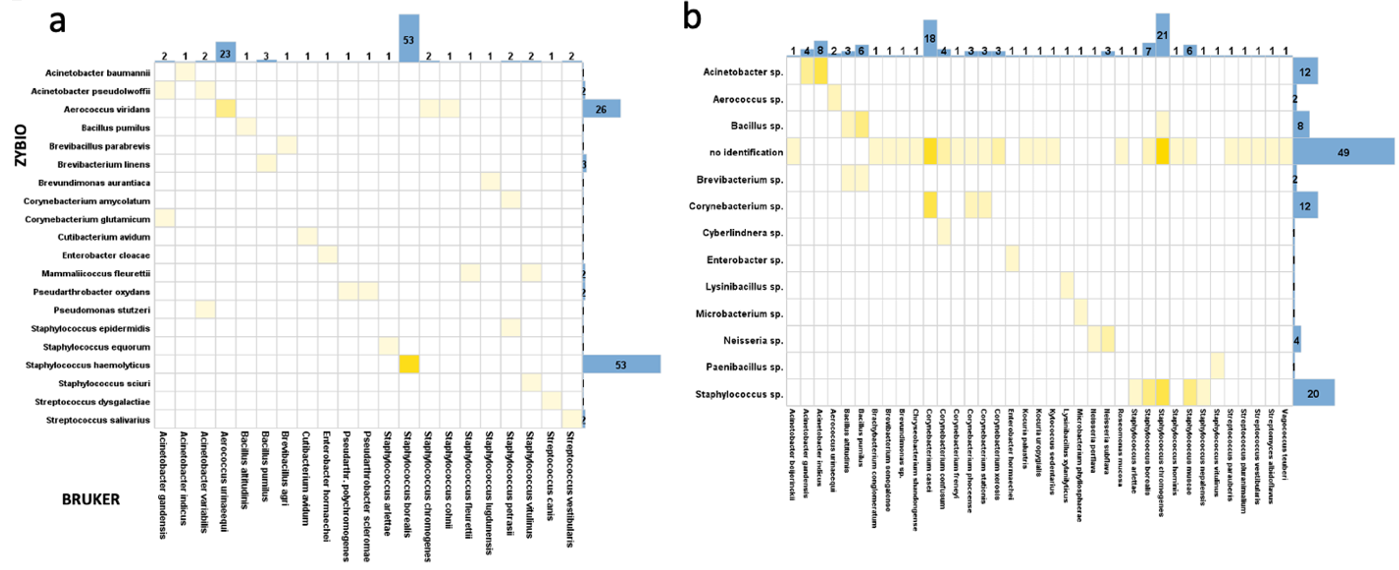


**Supplementary Figure 2** Species mismatch identification between the Bruker and Zybio systems. (A) Mismatch in species-level identification, (B) Mismatch of non-identification or genus-level identification. The intensity of the heat map color correlates with the number of samples identified by the two MALDI systems
